# Resolving Not to Quit: Evidence That Salient Group Memberships Increase Resilience in a Sensorimotor Task

**DOI:** 10.3389/fpsyg.2018.02579

**Published:** 2018-12-17

**Authors:** Jodie Green, Tim Rees, Kim Peters, Mustafa Sarkar, S. Alexander Haslam

**Affiliations:** ^1^Sport and Health Sciences, University of Exeter, Exeter, United Kingdom; ^2^Department of Sport & Physical Activity, Bournemouth University, Poole, United Kingdom; ^3^School of Psychology, The University of Queensland, Brisbane, QLD, Australia; ^4^Department of Sport Science, Nottingham Trent University, Nottingham, United Kingdom

**Keywords:** group memberships, performance, persistence, resilience, sensorimotor task, social identity

## Abstract

There is evidence that the social groups to which people belong can be a source of resilience in challenging times. In this paper, we examine whether social group memberships can also increase resilience in the face of negative performance feedback by encouraging task persistence. In two experiments (*N*s = 63, 61) participants completed three rounds of a performance task. In the experimental conditions (but not the control) participants were first asked to think about, and consider the importance of, either one or five important social groups of which they were members. In both experiments, participants who reflected on important social groups were more likely to persist in practicing the task after negative performance feedback than those in the control condition. In Experiment 2 only, there was also evidence of performance improvement after negative feedback for participants in experimental but not control conditions. There was no evidence that self-reported confidence, motivation, or self-efficacy accounted for the observed effects. Overall, this is the first study to provide evidence that salient group memberships can increase resilience in a sensorimotor task. Significantly, the findings suggest that groups are not just a context but also a critical psychological resource for performance following failure feedback.

## Introduction

As Thomas Edison so keenly observed, “Many of life’s failures are people who did not realize how close they were to success when they gave up.” Indeed, it is often small margins that separate failure from success. Bridging the gap is frequently a question of persistence: do we stick with our goals in the face of failure or do we give up? This dilemma has fascinated researchers across a number of related fields, including those interested in grit (Von Culin et al., 2014), optimism (Seligman, 2007), thriving (Porath et al., 2012), hardiness (Kobasa, 1979), and resilience (Coutu, 2002). Although exploration of individual qualities and character has underpinned much of the work in this area, recent research suggests that social factors may also have an important role to play. Notable among these social factors are people’s group memberships, which are known to act as a psychological resource in times of stress (Correll and Park, 2005; Iyer et al., 2009; Jones and Jetten, 2011; Steffens et al., 2016). In this paper, we explore the possibility that people’s membership of groups can help them increase their resilience in the face of negative performance feedback, and therefore that, all else being equal, social group memberships are a precursor to performance success.

Positive adaptation (e.g., persistence) in the face of adversity (e.g., obstacles, poor performance, and failure) is a hallmark of resilience, a topic examined across a range of contexts, including business (Gittell et al., 2006), education (Reis et al., 2005; Gu and Day, 2007), law enforcement (Miller, 2008), the military (Palmer, 2008; Reivich et al., 2011), medical services (Jackson et al., 2007), and sport (Galli and Vealey, 2008; Fletcher and Sarkar, 2012). At the heart of this work is the understanding that resilience is multifaceted and comprises a constellation of personal qualities that enable individuals to withstand the stressors they encounter in demanding contexts (Fletcher and Sarkar, 2012; Sarkar and Fletcher, 2014b). Although these qualities include having a positive and proactive personality, a sense of control, flexibility, adaptability, balance, and perspective, there is growing evidence that resilience also resides in people’s social connections and that, as Williams and Drury (Williams and Drury, 2009) observe, it is hard to demonstrate resilience on your own. For example, Fletcher and Sarkar (2012) found that the perception of available support from a variety of social agents was a factor that underpinned the stress-resilience-performance relationship in Olympic champions. Similarly, a study of 13 high achievers from a range of domains (including sport, medical services, and the creative arts) noted that perceptions of social support from others appeared to be a key ingredient of resilience (Sarkar and Fletcher, 2014a). Importantly, the benefits of social connections are not limited to a person’s individual relationships, as there is reason to believe that the *groups* to which people belong also matter.

Speaking to this point, a growing body of evidence suggests that people’s group memberships contribute to their ability to cope with and thrive in the context of challenging life circumstances (Sherman et al., 2007; Cruwys et al., 2014a; Jetten et al., 2014; Steffens et al., 2016). This work is situated in the social identity tradition (Tajfel and Turner, 1979, 1985), which argues that the groups to which people belong are central to their sense of self. That is, in the same way that people have a sense of who they are as a function of their unique individual characteristics (their personal identity), they also have a sense of who they are as a function of their membership of social groups (their social identity). As well as being an important factor underpinning team resilience in elite sport (Morgan et al., 2013, 2015, 2017), this sense of social identity has been shown to facilitate positive and constructive social interactions in a large range of other settings (Turner, 1991; Terry et al., 1999; Haslam, 2004; Greenaway et al., 2015b) and to be a basis for the provision and receipt of effective forms of social support (Haslam et al., 2012). This in turn suggests that people who have a positive sense of social identity in a performance setting (e.g., as a member of a sports or work team) are more likely to receive, and benefit from, the social support of other group members (Rees et al., 2015). Indeed, in their study of the 2003 England rugby union World Cup winning team, Morgan et al. (2015) proposed that “another possible explanation [for social identity processes facilitating team resilience] is that the team’s distinctive social identity provided a psychological basis for receiving—and gaining benefits from—the social support of team members” (p. 98).

Interestingly, there is evidence that merely making important group memberships salient can sometimes be enough to confer resilience in the face of performance-related setbacks. If correct, this suggests that a positive social identity may even help people to cope in circumstances where fellow group members are not present or able to provide immediate material assistance. To explore this possibility, Cruwys et al. (2014b) asked participants in experimental conditions to reflect on one or three important social groups before attempting to solve four unsolvable problems. Participants in a control condition did not complete the group reflection task. All participants were then provided with performance failure feedback (i.e., that they had not managed to solve any of the problems correctly). In line with their predictions, the researchers found that participants who reflected on either one or three important groups reported significantly lower levels of negative mood in response to the failure feedback than did participants in the control condition.

Although Cruwys and her colleagues found no evidence that reflecting on three groups was more beneficial than reflecting on just one group, there is in fact a growing body of work which suggests that, when it comes to group memberships, more is better. In particular, people with multiple positive group memberships report better levels of health and wellbeing than those with fewer. They also cope better with a range of life transitions—whether these are developmental or unexpected (Iyer et al., 2009; Jones et al., 2012).

Of specific relevance for this paper, there is also some evidence that membership of multiple groups may be beneficial in performance environments. In particular, Jones and Jetten (2011, Study 1) found that novice athletes’ resilience (measured via heart rate recovery) when learning to race bobsleigh, luge or skeleton was positively associated with the number of groups to which they belonged. Moreover, in a second study, Jones and Jetten asked participants to reflect on one, three or five important group memberships before immersing their hands in ice water for as long as possible. They found that participants who reflected on five groups were subsequently able to hold their hand in the water for longer than participants who reflected on one or three groups. This raises the possibility that resilience is more likely to be enhanced in performance environments the greater the number of group memberships made salient.

The findings reviewed above suggest that group memberships can play an important role in people’s resilience in the face of negative performance feedback. To the extent that this translates into a greater tendency to persist in task practice, this should lead to performance improvements (Newell and Rosenbloom, 1993; Krampe and Ericsson, 1996). This in turn suggests that one factor that may distinguish those who fail from those who push on and ultimately succeed is membership of social groups that provide a sense of positive social identity. In the present research, we adopted the approach of Jones and Jetten (2011) and Cruwys et al. (2014b) and sought to provide an experimental test of this idea by manipulating the salience of important social groups before asking participants to complete a golf-putting performance task in the face of negative performance feedback.

Our use of a golf putting task follows a long history of psychological research using motor tasks (Beilock and Carr, 2001; Rees and Freeman, 2009; Rees et al., 2013) and/or athletic samples (Jones and Jetten, 2011) to examine (social) psychological processes. Indeed, in their examination of heart rate recovery, Jones and Jetten (2011) used athletes from bobsleigh, luge, and skeleton to draw inferences about the impact of group memberships. Rees et al. (2013) used a dart-throwing task to examine aspects of ingroup influence and intergroup competition. More specific to the present studies, Beilock’s and Carr (2001) work on choking under pressure notes the benefits of using the complex sensorimotor task of golf-putting, because it lends itself well to providing discrete, objective, trial-by-trial assessments of performance accuracy. The often highly pressurized, visible, time-framed, and public nature of performance may well offer an excellent vehicle for examining social psychological processes. As Walsh (2014) argued in the context of testing the limits of current cognitive neuroscience, tasks such as ours offer “... a demanding activity requiring more cognitive skills than is often appreciated,” which may help us “... learn something about behavior relevant to the normal population” (Walsh, 2014).

The provision of negative performance feedback is an approach that has been commonly adopted in the sport resilience literature (Seligman et al., 1990; Martin-Krumm et al., 2003; Mummery et al., 2004). For example, Seligman et al. (1990) examined failure in university swimmers by falsely notifying them that their performance time was slower than their actual performance on an initial time trial. Those swimmers with an optimistic explanatory style then completed a subsequent trial in a time that was equal to, or faster, than their first. Martin-Krumm et al. (2003) extended this work, manipulating the beliefs of high school students, by informing them that they had not performed as well in a basketball task as others. Following failure feedback on the task, optimistic participants were more confident, less anxious, and performed better than their pessimistic counterparts.

In the present and first study of its type, we examined whether salient group memberships can increase resilience in a sensorimotor task. Using an experimental protocol, we randomly allocated participants either to a control condition (where no groups were made salient) or to one of two experimental conditions: a single group condition in which participants were asked to reflect on one important group, or a five group condition, where they instead reflected on five important groups. This allowed us to assess participants’ resilience (via persistence with, and performance on, the putting task following negative performance feedback) as a function of both the salience of social groups (i.e., control versus experimental conditions) and the *number* of salient social groups (i.e., one- versus five-groups conditions). More specifically, our hypotheses were as follows:

H1: Participants in the one- and five-groups conditions will show greater (a) task persistence and (b) performance following negative performance feedback than those in the control condition.

H2: Participants in the five-groups condition will show greater (a) task persistence and (b) performance following negative performance feedback than those in the one-group condition.

In this project, we also aimed to provide new insights by exploring potential mechanisms through which group membership salience might have these effects. Although there is limited evidence for the psychological mechanisms that mediate the influence of membership of multiple groups on outcomes [a notable exception is Cruwys et al. (2014b), who found evidence for a mediating role of depressive attributions in mood], authors have speculated that multiple group memberships promote resilience because they are a basis for psychological resources such as personal agency, self-affirmation, self-knowledge and self-efficacy (Jones and Jetten, 2011). In line with recommendations to examine the role of such factors (Jones and Jetten, 2011) in the present paper we sought to assess the mediating roles of three key variables that have been shown to underpin performance effects more generally: confidence, motivation, and self-efficacy (Galli and Vealey, 2008; Gucciardi, 2011; Fletcher and Sarkar, 2012).

## Experiment 1

### Methods

#### Participants

Participants were 63 university athletes (38 females, 25 males) with an average age of 20.37 years (*SD* = 1.52), who competed in a mix of team (*N* = 48: field hockey, netball, polo, rugby, soccer), individual (*N* = 2: karate, squash) and mixed individual/team (*N* = 13: badminton, dance, sailing, swimming, tennis) sports. The majority of participants reported having had very little (*N* = 42) or no (*N* = 17) golf putting experience (*N* = 3 reported moderate experience and *N* = 1 reported a lot of experience). The experiment received approval from the first author’s institutional human research ethics board, and participants granted their written informed consent.

#### Procedure

Participants were tested individually and randomly assigned to control, one-group and five-groups conditions (*N* = 21 in each condition). On arrival at the laboratory, participants were shown the materials for the putting task. These consisted of an artificial putting carpet that measured 490 cm long by 185 cm wide. The carpet had a putting marker and, 305 cm away, a target circle of 10 cm diameter. Participants were told that the aim of the task was to stand at the putting marker and putt the golf ball using a standard 90 cm right- or left-handed golf putter so that it stopped as close to the center of the target as possible. Participants were told that over the course of the experiment, they would complete three trials of six putts. Their performance in a given trial would be their average accuracy (measured as distance from the putted ball to the center of the target) across the six putts.

#### Baseline Measures and Performance

The baseline measures consisted of a baseline questionnaire, a baseline pre-trial questionnaire and the baseline practice and performance measures. Participants were first asked to complete a baseline questionnaire. This measured their *multiple group memberships* [a single item from Iyer et al. (2009): “I belong to multiple groups”; 1 = strong disagree, 7 = strongly agree], *social support* [four items from Haslam et al. (2005): e.g., “Do you get the help you need from other people?” 1 = not at all; 7 = completely] and current *affective state* [20-item Positive and Negative Affect Scale – PANAS – Watson et al. (1988): e.g., “Interested,” “Excited,” “Distressed,” “Scared,” 1 = very slightly or not at all, 5 = extremely].

After this, participants were asked to reflect on the upcoming putting performance trial and to respond to a baseline pre-trial questionnaire. This questionnaire measured their task *motivation* (two items: “It is important for me to do well on the task,” “I intend to put a lot of effort into this task”; 1 = not at all; 7 = extremely), *confidence* (two items adapted from the Competitive State Anxiety Inventory-2, Martens et al., 1990: “I’m confident about performing well,” “I’m confident about coming through under pressure,” 1 = not at all, 4 = very much so) and *self-efficacy* [following Bandura (2005), this assessed the maximum level of performance that they expected to attain from 1 = lowest performance to 10 = highest performance].

The baseline performance trial was preceded by a 2-min free practice period. Participants were told that they could use this time to practice their putting, read the magazines that had been provided for them, or simply have a rest (Le Foll et al., 2006, 2008). The experimenter left the room for the duration of this practice. Participants were filmed throughout the experiment and this footage was used to count the number of practice putts that participants took during the free-practice period. At the end of the practice period, the experimenter re-entered the room and started the baseline performance trial. After each of the six putts, the experimenter measured the distance (in cm.) between the golf ball’s final resting position and the center of the target. Participants’ performance for this baseline trial was the average distance across the six putts (where higher scores are indicative of less accurate putting).

### Group Salience Manipulation, Post-manipulation Measures and Performance

After the baseline practice and performance trials, group salience was manipulated for participants in the experimental conditions. These participants were told that the experimenters were interested in the groups to which they belonged. They were told that groups referred to any collective to which they belonged with other people, and that this could include groups related to work, university, hobbies and interests, social activities or sports. Participants in the one-group condition were then asked to think of one such group in their life, to describe its importance on a seven-item scale (“This group is important to me,” 1 = strongly disagree, 7 = strongly agree) and to reflect on the group’s importance, following instructions to “Now take a moment to think about your group. For a few moments, please take time to consider why your group is important or unimportant to you.” Participants in the five-groups condition were asked to complete this process for another four groups. The manipulation was delivered in a neutral way, so as to avoid any potential for participants to consider that the questions about social groups might be used to enhance performance. Thus, participants were unaware of the hypothesized effects for the manipulation. Furthermore, given that the manipulation was delivered after the baseline performance trial (and not after receiving performance feedback—see below), the integrity of the experimental design is assured.

#### Post-manipulation Pre-trial Measures

All participants (i.e., those in the control as well as experimental conditions) were then asked to complete a questionnaire about the upcoming performance trial. This post-manipulation pre-trial questionnaire was identical to the one administered before the baseline performance trial. Once participants had completed this questionnaire, the experimenter took them through the post-manipulation performance trial, measuring their performance as before.

#### Failure Feedback, Post-feedback Measures and Performance

Once participants had completed the post-manipulation performance trial, the experimenter provided them with negative performance feedback. This involved telling them that their putting performance across the two completed performance trials placed them in the bottom 30% of participants in the experiment so far (Coffee et al., 2009; Vine and Wilson, 2010). Participants were then asked to consider their upcoming (and final) performance trial before completing a post-feedback pre-trial questionnaire that was the same as that described in the previous section. This was followed by the post-feedback performance trial, which consisted of a 2-min free practice period (which provided the measure of task persistence) followed by the six performance putts. The procedures here were identical to those described at baseline.

#### Data Analysis

When testing our hypotheses, we accounted for baseline practice and accuracy differences by computing changes in later practice and accuracy variables from baseline. Our primary analytical approach thus consisted of running one-way between-subjects ANOVA (condition: control, one group or five groups) examining putting practice and performance accuracy relative to baseline over the course of the experiment. Where these tests revealed a significant effect of experimental condition, we ran contrasts that provided direct tests of H1 and H2. The H1 contrast (values: -2, 1, 1) compared the control and experimental conditions. The H2 contrast (values: 0, -1, 1) compared the one and five group conditions. Effect sizes for our main analyses were deemed small (0.01), medium (0.09) or large (0.25) for eta squared, and small (0.2), medium (0.5) or large (0.8) for Cohen’s *d*.

### Results

#### Scale Construction and Baseline Descriptives

We created scales by averaging relevant items. At baseline, this included scales of social support (α = 0.65), positive affect (α = 0.88), negative affect (α = 0.83), motivation (*r*_Spearman-Brown_ = 0.44), and confidence (*r*_Spearman-Brown_ = 0.65). Post-manipulation this included a measure of motivation (*r*_Spearman-Brown_ = 0.52) and confidence (*r*_Spearman-Brown_ = 0.64). Post-feedback this also included a measure of motivation (*r*_Spearman-Brown_ = 0.53) and confidence (*r*_Spearman-Brown_ = 0.70).

Participants in the three conditions did not differ in terms of age, *F*(2,60) = 0.13, *p* = 0.881, *η^2^=* 0.00, golf putting experience, *F*(2,60) = 0.54, *p* = 0.588, *η^2^=* 0.02, membership of multiple groups, *F*(2,60) = 1.09, *p* = 0.342, *η^2^=* 0.04, or social support, *F*(2,60) = 0.20, *p* = 0.823, *η^2^=* 0.01. They also did not differ at baseline in terms of positive affect, *F*(2,60) = 0.15, *p* = 0.860, *η^2^=* 0.01, negative affect, *F*(2,60) = 0.16, *p* = 0.853, *η^2^=* 0.01, or motivation, *F*(2,60) = 0.64, *p* = 0.530, *η^2^=* 0.02. Examining group differences with Bayes Factors (BF_10_ = 0.14–0.29, i.e., moderate evidence in favor of the null hypothesis over the alternative hypothesis) provided further support for these null effects. There were, however, significant differences in baseline confidence, *F*(2,60) = 7.99, *p* = 0.001, *η^2^=* 0.21, and self-efficacy, *F*(2,60) = 3.20, *p* = 0.048, *η^2^=* 0.10, such that one-group participants expressed higher confidence (*M* = 2.81, *SD* = 0.56) and self-efficacy (*M* = 7.05, *SD* = 2.0) at baseline than control participants (confidence *M* = 2.17, *SD* = 0.46, *d* = 1.25; self-efficacy *M* = 5.76, *SD* = 1.51, *d* = 0.73). Participants in the five-groups condition did not differ from those in the other conditions in terms of their confidence (*M* = 2.45, *SD* = 0.54) and self-efficacy (*M* = 6.86, *SD* = 1.77).

#### Putting Practice and Accuracy

Table 1 presents participants’ average number of practice putts and putting accuracy over the course of the experiment as a function of condition. As noted above, our analysis focused on running one-way between subjects ANOVA (condition: control, one group or five groups) on changes in practice and accuracy from baseline.

**Table 1:** Experiments 1 and 2 putting practice and accuracy—mean (*SD*).

Experiment	Measure	Trial	Control	One	Five
1	Practice Putts	Baseline	4.86 (3.10)	5.71 (3.59)	5.95 (3.04)
		Post-feedback	1.81 (2.50)	5.62 (3.76)	7.00 (3.30)
	Accuracy^a^	Baseline	55.43 (27.36)	58.23 (33.33)	73.00 (30.62)
		Post-manipulation	51.90 (16.60)	56.64 (30.44)	47.79 (19.37)
		Post-feedback	46.72 (25.96)	43.87 (14.46)	61.63 (34.55)
2	Practice Putts	Baseline	8.95 (2.72)	8.45 (1.39)	8.29 (1.79)
		Post-feedback	8.45 (2.58)	9.45 (3.76)	10.14 (2.95)
	Accuracy^a^	Baseline	49.58 (21.66)	53.24 (18.15)	55.77 (19.81)
		Post-manipulation	40.30 (14.39)	48.83 (11.77)	47.35 (17.94)
		Post-feedback	47.83 (20.39)	39.24 (11.40)	39.47 (13.19)


*^a^Performance accuracy measured as average distance (in cm) between golf ball and target over six putts.*

**FIGURE 1:**
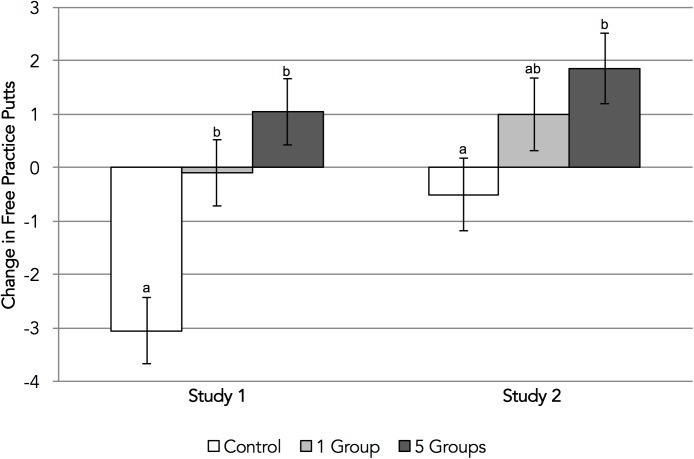
Experiments 1 and 2: changes in free putting practice from baseline. Error bars represent standard errors around condition means. Condition means with different letters significantly differ according to *post hoc* Tukey HSD tests.

The ANOVA testing the effect of group salience on putting practice revealed the expected significant effect of condition, *F*(2,60) = 11.78, *p* < 0.001, *η^2^=* 0.28—a large effect. As can be seen in Figure 1, participants in the group salience conditions were more likely to persist with their putting practice after negative performance feedback than those in the control condition. Next, we ran the specified contrast tests. In line with H1a, participants in the group salience conditions were significantly more likely to persist with their putting practice than participants in the control condition, *F*(1,60) = 21.83, *p* < 0.001, *d* = 1.25—a very large effect, implying over one standard deviation’s greater levels of persistence. However, H2a was unsupported as there was no evidence that participants in the five-groups condition persisted more on this task than participants in the one-group condition, *F*(1,60) = 1.72, *p* = 0.194, *d* = 0.41.

**FIGURE 2:**
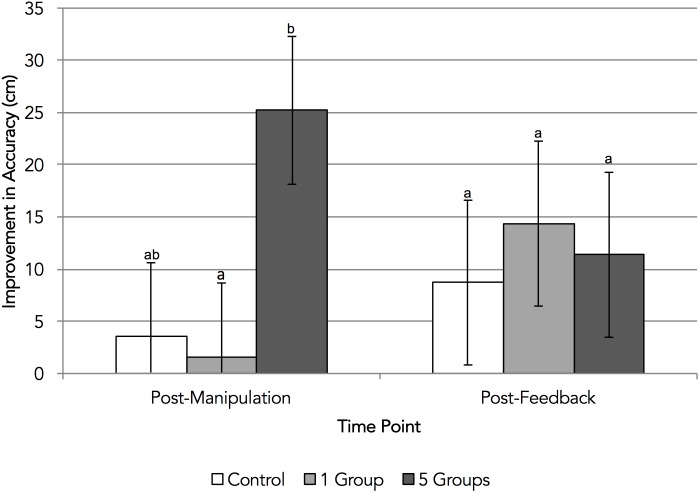
Experiment 1: change in putting accuracy from baseline. Error bars represent standard errors around condition means; bars that extend below the horizontal axis are not represented. Condition means with different letters significantly differ according to *post hoc* Tukey HSD tests.

Participants’ putting accuracy relative to baseline is summarized in Figure 2. We repeated this set of analyses to examine the effect of group salience on putting accuracy after negative performance feedback. Unexpectedly, the one-way ANOVA of the change in putting accuracy from baseline was not significant, *F*(2,60) = 0.13, *p* = 0.880, *η^2^=* 0.00. Therefore, there was no evidence to support H1b or H2b. We also repeated this analysis to examine the effect of group salience on the change in participants’ putting accuracy post-manipulation. This did reveal an effect of condition, *F*(2,60) = 3.43, *p* = 0.039, *η^2^=* 0.10—a moderate effect. Here, though, while the contrast between control and experimental conditions was not significant, *F*(1,60) = 1.30, *p* = 0.260, *d* = 0.30 the contrast between the one- and five-groups conditions was, *F*(1,60) = 5.57, *p* = 0.022, *d* = 0.73—a moderate effect, implying nearly three quarters of a standard deviation’s improvement in accuracy.

As can be seen in Figure 2, after the identity salience manipulation, participants in the five-groups condition appeared to show greater improvement in their accuracy than participants in the one-group condition. While this could point to the direct impact that making a larger number of groups salient has on performance, it is important to note that participants in the five-groups condition showed the least accurate performance at baseline (although this difference was not significant, *p* = 0.158), which may account for the relatively large improvement on the subsequent trial.

#### Sensitivity Analysis

To determine whether the inclusion of the one participant (from the one group condition) with “a lot of” golf-putting experience had a significant impact on the obtained results, we ran our main analyses with this participant omitted. Results for persistence [*F*(2,59) = 11.55, *p* < 0.001, *η^2^=* 0.28] and performance accuracy [*F*(2,60) = 0.14, *p* = 0.872, *η^2^=* 0.01] were largely unchanged.

#### Group Salience Mechanisms

To see whether the beneficial impact of the group salience manipulation on participants’ tendencies to persist in their putting practice after receiving negative performance feedback could be explained by any of the process variables, we calculated the changes in participants’ post-feedback motivation, confidence and self-efficacy from baseline. We then analyzed change in these measures by means of one-way ANOVA (condition: control, one group or five groups).

This analysis indicated that there was no effect of condition on post-feedback changes in participants’ motivation, *F*(2,60) = 0.74, *p* = 0.484, *η^2^=* 0.02, or self-efficacy, *F*(2,60) = 0.60, *p* = 0.552, *η^2^=* 0.02. However, there was a significant effect of condition on post-feedback changes in participants’ confidence, *F*(2,60) = 4.67, *p* = 0.013, *η^2^=* 0.14 (this latter result remained significant after applying a Bonferroni correction for the three tests: i.e., α = 0.017). *Post hoc* analyses indicated that participants in the control condition showed a smaller decline in confidence over the course of the experiment (*M* = 0.14, *SD* = 0.57) than participants in the one-group condition (*M* = -0.38, *SD* = 0.52; Tukey HSD *p* = 0.014, *d* = 0.96) or (marginally) the five-groups condition (*M* = -0.26, *SD* = 0.64; Tukey HSD *p* = 0.071, *d* = 0.66). For completeness, repeating this analysis for changes in participants’ post-manipulation motivation, confidence and self-efficacy from baseline demonstrated no effect of condition on post-manipulation changes in participants’ motivation, *F*(2,60) = 1.98, *p* = 0.147, *η^2^=* 0.06, or self-efficacy, *F*(2,60) = 2.63, *p* = 0.080, *η^2^=* 0.08. However, there was a significant effect of condition on post-manipulation changes in participants’ confidence, *F*(2,60) = 4.70, *p* = 0.013, *η^2^=* 0.14 (this latter result remained significant after applying a Bonferroni correction for the three tests: i.e., α = 0.017). *Post hoc* analyses indicated that participants in the control condition demonstrated a marked increase in confidence from baseline (*M* = 0.45, *SD* = 0.57) compared with participants in the one-group condition (*M* = 0.00, *SD* = 0.47; Tukey HSD *p* = 0.014, *d* = 0.86) and (marginally) the five-groups condition (*M* = 0.10, *SD* = 0.46; Tukey HSD *p* = 0.064, *d* = 0.68). Mediation analysis (Hayes and Preacher, 2014) showed little evidence that the post-feedback confidence effect could account for the group differences in persistence—the confidence intervals around the indirect effects of the group salience manipulations (versus control) on persistence through post-feedback confidence included zero: one group -0.08, CI [-0.97, 0.68]; five groups -0.06, CI [-0.79, 0.51]. There was also no influence of the other two potential group salience mechanisms, with the confidence intervals around the indirect effects of the group salience manipulations (versus control) on persistence through post-feedback motivation and self-efficacy including zero. Repeating this analysis for participants’ post-feedback performance did not reveal any significant effects either.

### Discussion

In line with H1a, this experiment found that participants in conditions that made group membership salient were significantly more likely to demonstrate resilience (i.e., persist with their practice on a putting task after negative performance feedback) than participants in a control condition, whose persistence markedly decreased. Although descriptively this effect appeared to be most pronounced for participants in the five-groups condition, the difference between the one- and five-groups conditions was not significant. Accordingly, there was no support for H2a.

There was no evidence that making group memberships salient led to improved post-feedback performance (as asserted by H1b and H2b). Thus, the increased post-feedback practice that we observed for these participants did not translate into more accurate putting. Unexpectedly, though, participants in the five-groups condition showed a significantly greater improvement in accuracy from baseline after the group salience manipulation than participants in the one-group condition. While this could indicate an immediate performance improvement associated with making a greater number of important social groups salient, we do not have any theoretical basis for expecting such a direct benefit. Rather, it seems likely that the relatively poor performance of participants in the five-groups condition at baseline meant that they had the greatest potential for subsequent improvement.

In Experiment 2, we sought to directly replicate this study to see whether we could reproduce our findings. The only substantial change to the experimental procedure involved removing one of the baseline assessments (social support, for which scale reliability was sub-optimal), and using a different putting surface (see below).

## Experiment 2

### Methods

#### Participants

Participants were 61 university athletes (30 females, 31 males) with an average age of 20.82 years (*SD* = 1.20), who competed in a mix of team (*N* = 39: basketball, field hockey, netball, polo, rugby, soccer, volleyball), individual (*N* = 8: boxing, golf, karate, kickboxing, squash) and mixed individual/team (*N* = 14: athletics, badminton, dance, gymnastics, rowing, sailing, swimming, tennis) sports. The majority of participants reported having had either very little (*N* = 27) or no (*N* = 23) prior golf putting experience (another 8 respondents reported moderate levels of experience and 3 reported having a lot). This experiment received ethics approval from the first author’s institutional human research ethics board, and participants granted their written informed consent.

#### Procedure

Participants were recruited individually, and on arrival at the laboratory were randomly allocated to one of the three conditions: control (*N* = 20), one group (*N* = 20) or five groups (*N* = 21). The experiment’s procedure was largely identical to that of Experiment 1. This time, however, participants performed the task on an artificial putting surface that was 600 cm long and 213 cm wide (in Experiment 1, participants putted on carpet; as this allowed the ball to roll with less natural resistance, it was associated with greater variability in accuracy). As before, the distance between the putting marker and the target (an “X”) was 305 cm. The questionnaire measures were also simplified so that at baseline they only assessed affective state (with the PANAS), motivation, confidence and self-efficacy; pre-trial scales were as described in Experiment 1.

### Results

#### Scale Construction and Baseline Descriptives

Scales were created by averaging relevant items. At baseline, there were scales of positive affect (α = 0.88), negative affect (α = 0.68), motivation (*r*_Spearman-Brown_ = 0.48), and confidence (*r*_Spearman-Brown_ = 0.77). Post-manipulation, there were scales of motivation (*r*_Spearman-Brown_ = 0.69) and confidence (*r*_Spearman-Brown_ = 0.91). Post-feedback there were also scales of motivation (*r*_Spearman-Brown_ = 0.77) and confidence (*r*_Spearman-Brown_ = 0.86).

Participants in the three conditions did not differ in age, *F*(2,58) = 3.02, *p* = 0.057, *η^2^=* 0.09, golf putting experience, *F*(2,58) = 0.20, *p* = 0.820, *η^2^=* 0.01, or membership of multiple groups, *F*(2,58) = 1.02, *p* = 0.366, *η^2^=* 0.03. They also did not differ at baseline in positive affect, *F*(2,58) = 0.31, *p* = 0.739, *η^2^=* 0.01, negative affect, *F*(2,58) = 0.43, *p* = 0.655, *η^2^=* 0.01, motivation, *F*(2,58) = 0.13, *p* = 0.877, *η^2^=* 0.01, confidence, *F*(2,58) = 0.04, *p* = 0.958, *η^2^=* 0.00, or self-efficacy, *F*(2,58) = 1.53, *p* = 0.225, *η^2^=* 0.05. Examining group differences with Bayes Factors (BF_10_ = 0.14-0.41, i.e., moderate to anecdotal evidence in favor of the null hypothesis over the alternative hypothesis) generally provided further support for these null effects. There was one exception in the case of age (BF_10_ = 1.22, i.e., anecdotal evidence in favor of the alternative hypothesis), primarily attributable to a lower age for the five-group condition (*M* = 20.33, *SD* = 0.80) compared to the one-group condition (*M* = 21.20, *SD* = 1.24).

#### Putting Practice and Accuracy

Table 1 presents participants’ average number of practice putts and putting accuracy over the course of the experiment as a function of condition. As in Experiment 1, when testing our hypotheses, we accounted for baseline practice and accuracy differences by computing changes in these variables from baseline. As before, we examined the impact of making groups salient on putting practice and accuracy with one-way between-subjects ANOVA (condition: control, one group or five groups). Where these were significant, we directly tested H1 and H2 by using contrasts that compared the control and experimental conditions (contrast values: -2, 1, 1) and the one-group and five-groups conditions (contrast values: 0, -1, 1).

When examining participants’ post-feedback putting practice, we found the expected significant effect of condition, *F*(2,58) = 3.16, *p* = 0.050, *η*^2^= 0.10—a moderate effect. As can be seen in Figure 1, participants in the group salience conditions were more likely to persist with putting practice than those in the control condition. Supporting this observation, and providing further evidence for H1a, the associated contrast (comparing control and experimental conditions) was significant, *F*(1,58) = 5.43, *p* = 0.023, *d* = 0.64—a moderate effect, implying over half a standard deviation’s increase in persistence. However, the contrast between the one-group and five-groups conditions was not significant, *F*(1,58) = 0.82, *p* = 0.369, *d* = 0.28, and there was therefore no support for H2a. These findings directly replicate those of Experiment 1.

**FIGURE 3:**
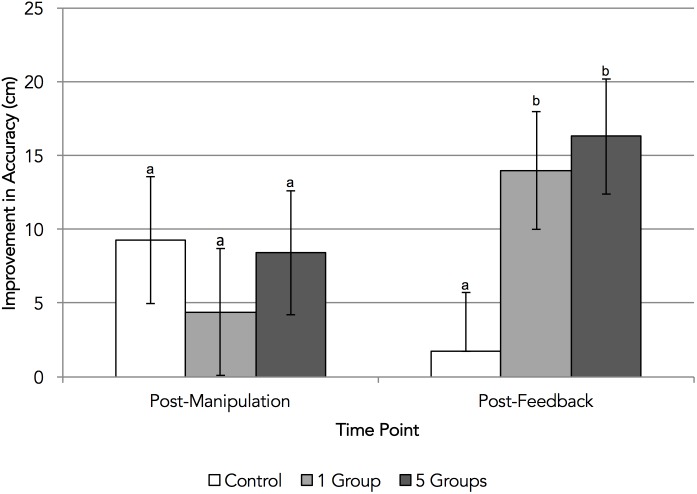
Experiment 2: change in putting accuracy from baseline. Error bars represent standard errors around condition means; bars that extend below the horizontal axis are not represented. Condition means with different letters significantly differ according to *post hoc* Tukey HSD tests.

Next, we analyzed participants’ post-feedback putting accuracy and this revealed the expected main effect of condition, *F*(2,58) = 3.89, *p* = 0.026, *η*^2^= 0.12—a moderate effect. Unlike Experiment 1, then, there was evidence that the group salience manipulation had affected both persistence and performance. As can be seen in Figure 3, the post-feedback putting accuracy of participants in the group salience conditions improved more than that of participants in the control condition, *F*(1,58) = 7.58, *p* = 0.008, *d* = 0.75—a moderate effect, implying three quarters of a standard deviation’s improvement in accuracy—thereby supporting H1b. However, the contrast between the one- and five-groups conditions was not significant, *F*(1,58) = 0.17, *p* = 0.681, *d* = 0.13, and so there was no support for H2b.

Also unlike Experiment 1, repeating this analysis for participants’ *post-manipulation* putting accuracy (see Figure 3) did not reveal any significant effect of condition, *F*(2,58) = 0.37, *p* = 0.695, *η^2^=* 0.01—a small effect. Therefore, there was no evidence in this experiment of a direct effect of the group salience manipulation on performance. Rather, making groups salient appeared to confer performance benefits only in the context of a challenge in the form of negative performance feedback.

#### Sensitivity Analysis

To determine whether the inclusion of the three participants (one from each condition) with “a lot of” golf-putting experience had a significant impact on the obtained results, we ran our main analyses with these participants omitted. Results for persistence [*F*(2,55) = 3.48, *p* = 0.038, *η^2^=* 0.11] and performance accuracy [*F*(2,55) = 4.30, *p* = 0.018, *η^2^=* 0.14] were largely unchanged.

#### Practice and Performance

To establish whether participants’ persistence in their putting practice after receiving negative performance feedback accounted for the impact of the group salience manipulations on post-feedback performance, we ran a mediation analysis (Hayes and Preacher, 2014) with contrast coding for the group salience manipulation variable. Our model used the change in post-feedback accuracy from baseline as the dependent variable; change in post-manipulation accuracy from baseline was included as a covariate. Our proposed mediator was change in post-feedback practice. Across conditions, there was a marginal association between post-feedback putting persistence and subsequent improvements in accuracy (*b* = -1.19, *p* = 0.059). The confidence interval around the indirect effect of the group salience manipulations (versus control) on post-feedback improvements in accuracy through putting persistence did, however, include zero -2.21, CI [-8.92, 0.02]. There was thus little evidence that the observed improvements in accuracy in the group salience conditions resulted from participants’ increased tendencies to persist in putting practice following negative performance feedback. Instead, it appears that the group salience manipulations had independent direct effects on persistence and performance.

#### Group Salience Mechanisms

To see whether the impact of the group salience manipulation on participants’ post-feedback putting persistence and improved accuracy could be explained by any of the process variables that we measured, we calculated changes in participants’ post-feedback motivation, confidence and self-efficacy from baseline. We analyzed these change measures by means of one-way between-subjects ANOVA (condition: control, one group or five groups). There was no effect of condition on post-feedback changes in participants’ self-reported motivation, *F*(2,58) = 0.82, *p* = 0.446, *η^2^=* 0.03, confidence, *F*(2,58) = 1.23, *p* = 0.299, *η^2^=* 0.04, or self-efficacy, *F*(2,58) = 0.46, *p* = 0.636, *η^2^=* 0.02. For completeness, repeating this analysis for changes in participants’ post-manipulation motivation, confidence and self-efficacy from baseline demonstrated no effect of condition on post-manipulation changes in participants’ self-reported motivation, *F*(2,60) = 0.26, *p* = 0.775, *η^2^=* 0.01, confidence, *F*(2,58) = 0.92, *p* = 0.404, *η^2^=* 0.03, or self-efficacy, *F*(2,58) = 0.01, *p* = 0.994, *η^2^=* 0.00. As in Experiment 1, there was no evidence of mediation (Hayes and Preacher, 2014) in relation to persistence or post-feedback performance.

### Discussion

Providing further support for H1a, this experiment replicated a key finding from Experiment 1—again showing participants whose group memberships had been made salient were subsequently more likely to demonstrate resilience (i.e., persist with putting practice after receiving negative performance feedback) than participants in a control condition. Importantly too, unlike Experiment 1, we obtained support for H1b as the putting accuracy of participants in the group salience conditions improved more after negative feedback than did that of participants in the control condition.

As in Experiment 1, however, there was no support for H2, as making five groups salient was no more beneficial—either for participants’ persistence or their performance—than making one group salient. Thus, while there was a tendency for participants in the five-groups condition to be more persistent and more accurate after negative feedback than those in the one-group condition, the differences were not significant.

Although we observed the same pattern of findings for participants’ putting persistence and performance accuracy, we did not find any evidence of an indirect effect of our experimental manipulation on accuracy via persistence. Accordingly, it appears that making groups salient had identical direct effects on persistence and performance. It is possible, however, that such a mediated effect only manifests itself over a long time period, and that the time-frame of the present paradigm was too short for persistence to pay off in the form of improved performance. Finally, like Experiment 1, this study could not find evidence for the psychological mechanisms through which group salience had its impact.

## General Discussion

The aim of the present research was to examine the capacity for people’s group memberships to function as a psychological resource that helps them to demonstrate resilience (i.e., persist in the face of negative performance feedback). Our findings were generally consistent with this possibility. In particular, in both experiments we found that participants who had been asked to reflect on either one or five important groups in their lives appeared more likely to persist in practicing their putting after being told that their performance was worse than that of most of their peers—thereby supporting H1a. In Experiment 2 (though not Experiment 1), these same participants also showed greater improvement in their putting performance—thereby supporting H1b. Somewhat unexpectedly, making groups salient appeared to have independent direct effects on persistence and performance, as there was little evidence that persistence fed into subsequent performance. Together, these findings support claims that the benefits of positive social identities may not be limited to contexts in which group members can provide immediate material assistance and support (Haslam et al., 2005; Rees et al., 2015). Instead, merely bringing important groups to mind may be enough for them to promote resilience.

These experiments could not, however, provide any evidence for the claim (articulated in H2) that making more (rather than fewer) groups salient would lead to more benefits. That is, there was little evidence that persistence and performance were any greater when participants were asked to bring five groups to mind rather than just one. While this aligns with the findings of Cruwys et al. (2014b), it is inconsistent with a body of work that points to the positive association between the number of groups that a person belongs to and their ability to adapt to difficult life transitions (Iyer et al., 2009; Jones et al., 2012; Cruwys et al., 2013). This may be viewed as unsurprising, given the relatively narrow focus of the present task (when compared with the life transitions work, noted above), such that more groups might not be expected to be of greater benefit. However, it is also inconsistent with the similarly narrow experimental findings of Jones and Jetten (2011). In light of this, it is worth noting that in the current experiments the impact of group salience on persistence was descriptively greatest in the five-groups condition (although this was less evident when looking at performance). It is possible, therefore, that experiments with larger sample sizes, and employing different tasks and contexts, are needed to expose differences between the one-group and five-groups conditions.

An additional aim in this paper was to explore the role of potential psychological processes that could account for the benefits of group salience on persistence and performance. To this end, we measured a number of processes that have been shown to underlie people’s behavior and performance in a range of domains, namely confidence, motivation and self-efficacy. However, we found very little evidence that participants’ experiences of these psychological states varied between conditions over the course of the experiments. Furthermore, there was little evidence that these factors could account for the observed changes in behavior. One reason for this might be that we simply failed to measure the psychological factor(s) that account(s) for the observed behavioral effects. In this respect, future work might consider whether other processes—such as belonging, adjustment, control, meaning-making, and purpose (Iyer et al., 2009; Greenaway et al., 2015a)—might be implicated in these. Another possibility, of course, is that the mechanisms that underlie these effects are not amenable to conscious introspection. Finally, as noted below, our sample sizes may not have been large enough to detect relevant process effects.

### Limitations and Future Research

Like any piece of research, these experiments have limitations. Of these, the most significant is their relatively small sample size. Although our achieved power in Experiment 1 for participants’ putting persistence was 0.99, in Experiment 2, our achieved power for the effect of group salience on participants’ putting persistence was only 0.62 and on performance was 0.71. In future research it would therefore be advisable to have much larger sample sizes, especially to provide a more definitive test of H2, and to aid power to observe potential mediated effects. Additionally, given the marginal association between post-feedback putting persistence and subsequent improvements in accuracy observed in Experiment 2, extended free practice time might also provide greater opportunity for subsequent improvement in accuracy and allow us to observe mediated effects.

We should also point out that scale reliability was sub-optimal for the social support scale in Experiment 1 (α = 0.65) and for the negative affect scale in Experiment 2 (α = 0.68). Nonetheless, because these were baseline assessments, used only to examine any potential initial group differences, these low reliabilities did not affect our main study analyses. However, Spearman–Brown reliability estimates for our two-item assessment of motivation (one of our process assessments) were sub-optimal throughout Experiment 1 (0.44, 0.52, and 0.53), and at baseline (0.48) and post-manipulation (0.69) in Experiment 2. Although we saw no differences between conditions for any of these assessments, this raises the possibility that our two-item scales should include more items in future research. Similarly, Spearman–Brown reliability estimates for our two-item assessment of confidence were sub-optimal in Experiment 1 at baseline (0.65) and post-manipulation (0.64). Our observed baseline differences in confidence should thus be treated with caution.

As we noted in the Introduction, our use of a sensorimotor (golf-putting) task following negative performance feedback can have implications for resilience effects more widely, but the use of a university-student sample with little golf-putting experience may limit the generalizability of our findings. Although the current study offers an initial and important examination of the impact of social group memberships on resilience in a sensorimotor task, future research should examine the link between group membership and resilience using different samples and contexts (e.g., experienced athletes performing stressful tasks in ‘real-world’ settings). Indeed, given the challenges associated with creating high levels of pressure in laboratory-based environments, future research is encouraged to explore the current study’s findings with elite athletes in top-level competition.

We should note that some changes between experiments may have influenced our results. The change in Experiment 2 to an artificial grass putting surface was associated with more consistent scores across participants (i.e., smaller standard deviations around performance accuracy measurements); it was also associated with notable increases in the number of practice putts. We can only speculate as to why there may have been such differences. One possibility is that the higher-quality putting surface and thus more professional appearance may have encouraged greater engagement with the experimental task. The change in experimenters across experiments might also have influenced the results. Regarding the latter point, there is recent evidence of the ways in which identification with experimenters can encourage participants to behave in ways that support their research goals (Van Bavel, 2018). In the present case, though, it is unclear how this process would explain between-condition differences of the form we observed. Nevertheless, the role that shared identity with an experimenter plays in producing practice and performance effects is certainly an interesting issue to explore in future research.

### Conclusion

Most people who succeed have experienced failure. As Edison intimated, this means that while failure (or poor performance) is not necessarily diagnostic of future success, giving up in the face of these challenges is. And yet while it might be easy for a golfing champion like Jack Niklaus to advise us that we should “resolve never to quit, never to give up, no matter what the situation,” it is far harder to know where the source of such resolve might lie. In the present experiments, we explored and found support for one potential answer—that it is our internalized group memberships that help us to demonstrate resilience (i.e., persist and potentially perform better) in the face of challenge (i.e., negative performance feedback) and thereby increase our chances of ultimate success. In this, then, we see that groups are not just a context but also a critical psychological *resource* for resilience and performance.

## Ethics Statement

This study was carried out in accordance with the recommendations of the University of Exeter Human Research Ethics Board, with written informed consent from all subjects. All subjects gave written informed consent in accordance with the Declaration of Helsinki. The protocol was approved by the University of Exeter Human Research Ethics Board.

## Author Contributions

JG, TR, KP, and SH designed the experiments. JG collected the data for Experiment 1. TR collected the data for Experiment 2. TR, KP, and JG analyzed the data. JG produced a draft of Experiment 1. TR and KP wrote the manuscript. SH made extensive comments on the manuscript. JG and MS provided feedback on the manuscript.

## Conflict of Interest Statement

The research was funded in part by the England and Wales Cricket Board. There are no patents, copyrights, products in development, or marketed products to declare. This does not alter our adherence to all the *Frontiers* policies on sharing data and materials.
